# Use of Pharmacogenetics to Optimize Immunosuppressant Therapy in Kidney-Transplanted Patients

**DOI:** 10.3390/biomedicines10081798

**Published:** 2022-07-26

**Authors:** Valentina Urzì Brancati, Carmelo Scarpignato, Letteria Minutoli, Giovanni Pallio

**Affiliations:** 1 Department of Clinical and Experimental Medicine, University of Messina, Via C. Valeria, 98125 Messina, Italy; valeurzi@hotmail.it (V.U.B.); gpallio@unime.it (G.P.); 2 Department of Health Sciences, United Campus of Malta, MSD 2080 Msida, Malta; carmelo.scarpignato@gmail.com; 3 Faculty of Medicine, Chinese University of Hong Kong, ShaTin, Hong Kong, China

**Keywords:** pharmacogenetics, polymorphism, SNP, cyclosporine, tacrolimus, mycophenolic acid, sirolimus, everolimus, kidney transplant

## Abstract

Immunosuppressant drugs (ISDs) are routinely used in clinical practice to maintain organ transplant survival. However, these drugs are characterized by a restricted therapeutic index, a high inter- and intra-individual pharmacokinetic variability, and a series of severe adverse effects. In particular, genetic factors have been estimated to play a role in this variability because of polymorphisms regarding genes encoding for enzymes and transporters involved in the ISDs pharmacokinetic. Several studies showed important correlations between genetic polymorphisms and ISDs blood levels in transplanted patients; therefore, this review aims to summarize the pharmacogenetics of approved ISDs. We used *PubMed* database to search papers on pharmacogenetics of ISDs in adults or pediatric patients of any gender and ethnicity receiving immunosuppressive therapy after kidney transplantation. We utilized as search term: “cyclosporine or tacrolimus or mycophenolic acid or sirolimus or everolimus and polymorphism and transplant”. Our data showed that polymorphisms in CYP3A5, CYP3A4, ABCB1, and UGT1A9 genes could modify the pharmacokinetics of immunosuppressants, suggesting that patient genotyping could be a helpful strategy to select the ideal ISDs dose for each patient.

## 1. Introduction

Allograft transplantation is the best treatment for organ end-stage failure, and a subsequent immunosuppressant drugs (ISDs)-based therapy is routinely used to prevent graft rejection. ISDs, however, are drugs with a narrow therapeutic window. Large inter/intra-patient variability both in pharmacokinetics (PK) and pharmacodynamics (PD) may lead to severe toxicity or lack of efficacy [1]. Amongst the immunosuppressant drugs, tacrolimus, cyclosporine, mycophenolic acid, everolimus, and sirolimus are the most commonly used for organ transplants [2].

Tacrolimus (TAC), also known as FK-506, is an immunosuppressant that belongs to the class of calcineurine (CNI) inhibitor, and it is available in two formulations: the immediate-release formulation (given twice daily) and the prolonged-release Tac (given once daily). In blood, the main reservoir for TAC are erythrocytes, but the percentage of the drug associated with these cells varies widely, and this variability seems to be due to inter-patient differences in hematocrit, the drug-binding capacity of erythrocytes, and concentration-dependent distribution of the drug between blood and plasma [3]. The metabolization of TAC takes place mostly in the liver and gut wall due to CYP3A4 and CYP3A5, with a minimal contribution of CYP3A7.

Cyclosporine (CsA) is a lipophilic cyclic peptide formed by 11 amino acids produced by the fungus *Typocladium inflatum,* and together with TAC, it belongs to the class of CNI-inhibitor. After entering the small-bowel epithelial cells, part of CsA is transported back into the lumen by an active mechanism of transport mediated by membrane-bound P-glycoprotein 1. CsA is metabolized in the liver mainly by CYP3A4 and in a lesser extent by CYP3A5; more than 30 metabolites have been described, and more than 90% of these metabolites are excreted in the bile [4].

Mycophenolic acid (MPA) is a potent, selective, reversible, and non-competitive inhibitor of inosine monophosphate dehydrogenase (IMPDH) type II, an enzyme implicated in de novo guanosine nucleotide synthesis, which is necessary for lymphocyte proliferation [5]. Therefore, the inhibition of this pathway prevents lymphocytes proliferation and T-cell activation. MPA is mainly metabolized in the liver, intestine, and kidney by UDP-glucuronosyltransferases (UGT). A major fraction is converted to the inactive 7-O-glucuronide (MPAG), and a minor fraction is converted to the active acyl glucuronide (AcMPAG) [6].

Everolimus (EVE) and sirolimus (SIR) act by inhibiting the response to IL-2, blocking the activation of T and B cells. This is obtained by their binding the cytosolic protein FK-binding protein 12 (FKBP12) forming a complex that inhibits the mammalian target of rapamycin (mTOR) pathway by directly binding the mTOR Complex1 (mTORC1) [7]. The metabolization of EVE and SIR take place mostly in the liver and gut wall due to CYP3A4 and CYP3A5.

The inter- and intra-individual variability observed in transplant recipients treated with ISDs can be due to multiple factors, such as age, body weight, hematocrit, hepatic and renal function, interaction with other drugs, comorbidities, and polymorphisms in genes involved in the metabolism or transporter of these drugs [8,9]. In particular, genetic factors have been estimated to account for an important part of inter-individual differences in drug metabolism and transplant outcomes.

The most important class of enzymes involved in the metabolism of ISDs is the CYP3A subfamily. In humans, there are four different CYP3A isoenzymes: CYP3A4, CYP3A5, CYP3A7, and CYP3A43 [10]. For ISDs, genetic polymorphisms of CYP3A4 and CYP3A5 isoenzymes are a significant part of the inter-individual variability observed within patients. Regarding CYP3A5, a single-nucleotide polymorphism (SNP) at position 6986 (6986A>G) is the most studied. Carriers of at least one copy of nucleotide A have *1 allele and are defined CYP3A5 expressers, while homozygotes G/G are *3/*3 and are considered non-expressers [11] because the substitution of G with A causes an altered mRNA splicing responsible for an early stop codon that produces a non-functional protein [12]. Therefore, CYP3A5 expressers may have a higher drug-metabolizing ability that could lead to therapeutic failure [13].

In regards to CYP3A4, the main polymorphism implicated in the metabolism of ISDs occurs at position 392 and is an A>G substitution producing a variant allele (CYP3A4*1B) with increased enzymatic activity [14]. In the same gene, another important polymorphism is a C>T substitution in the position 15,389 called CYP3A4*22, that results in low hepatic CYP3A4 mRNA expression and decreased CYP3A4 activity [15].

Another class of enzymes involved in inter-patient variability are the microsomal electron transfer flavoprotein Cytochrome P450 oxidoreductase (POR), which have been seen influencing CYP3A activity [16]. Human POR is highly polymorphic, and a 1508C>T substitution in POR gene results in an increased metabolism in CYP3A5 expresser [17].

However, inter- and intra-patient variability cannot be entirely explained with the polymorphisms in genes encoding for these two classes of enzymes, and therefore, other genes must be involved [18].

Thus, polymorphisms in transporters could be associated with variations in ISDs levels. Glycoprotein (P-glycoprotein, P-gp), which belongs to the family of the ATP binding cassette (ABC) membrane transporter, is encoded by multidrug-resistance gene 1 (MDR1), which is a very polymorphic gene, with about 100 SNPs identified in the coding region [19], and among these, the C3435T, G2677T/A, and C1236T are the most important for ISDs pharmacokinetics [20]. Another transporter involved in the pharmacokinetic profile of ISDs is multidrug-resistance-associated protein 2 (MRP2), encoded by the ABCC2 gene. In particular, a 24C>T, 1249G>A, 3972T>C substitution (rs717620, rs2273697, rs3740066) is frequently associated with altered ISDs blood levels [21]. Moreover, a large part of the PK of ISDs is determined by polymorphisms in the organic anion transporter (OAT) polypeptide proteins OATP1B1 and OATP1B3, coded by the SLCO1B1 and SLCO1B3 genes, respectively. They are influx transporters present on the apical side of hepatocytes and act as acellular entry gates important for the elimination by hepatic metabolism and biliary excretion [22].

Finally, polymorphisms in IMPDH2 genes that encodes for the target protein of mycophenolic acid and in UGT1A9 mainly involved in the metabolism of MPA may be responsible for the intra- and inter-individual variability of MPA levels [23,24].

For all these reasons, the aim of this review was to investigate the effects of several polymorphisms in genes involved in metabolism or transport of ISDs and their influence on the levels of these drugs.

## 2. Material and Methods

**Figure 1:**
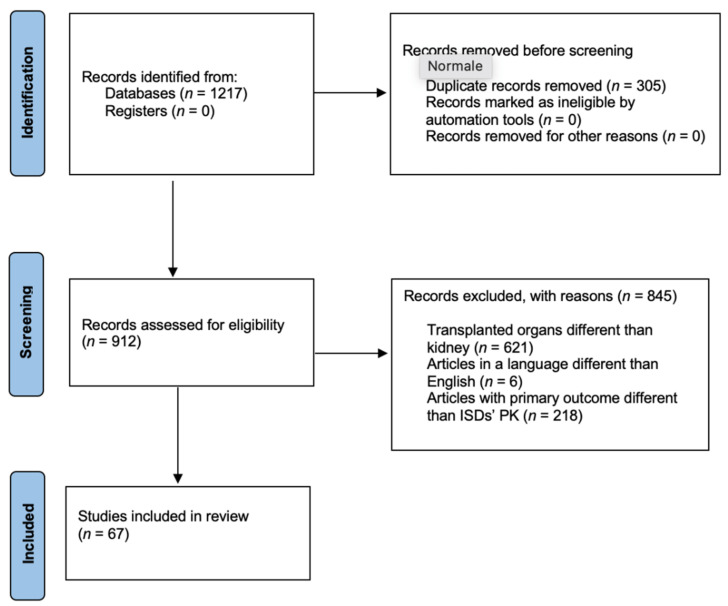
Flow diagram of literature search.

The database used to retrieve the papers was *PubMed*, and the following the search terms were used: “cyclosporine and polymorphism and transplant”; “tacrolimus and polymorphism and transplant”; “mycophenolic acid and polymorphism and transplant”; “sirolimus and polymorphism and transplant”; and “everolimus and polymorphism and transplant”. Papers on adults or pediatric patients of any gender and ethnicity receiving immunosuppressive therapy after transplant were included. Studies were excluded if: (i) immunosuppressant were used to treat other diseases, (ii) articles were written in a language other than English, (iii) papers were about unspecified genotypes, (iv) transplanted organs were other than kidney, and (v) primary outcomes were other than ISDs’ PK. The summary of the literature search is shown in Figure 1.

## 3. Results

### 3.1. Pharmacogenetics of Tacrolimus

In the context of immunosuppressive therapy after solid organ transplant, several studies have explored the role of SNPs in cytochromes. In particular, tacrolimus pharmacokinetics is mainly affected by polymorphisms in CYP3A4 and CYP3A5 genes. In this regard, Cheung et al. studied a population of 86 adult Chinese kidney transplant patients and found that CYP3A5 expressers needed a higher TAC dose compared with the nonexpressers [25]. Similar results were shown in several other studies conducted in different ethnic groups and post-transplant time periods [26,27,28,29,30] and by Muller et al., who also found that CYP3A5*3/*3 carriers showed higher inter-patient variability than CYP3A5*1/*1 and *1/*3 carriers [31]. Similarly, Gervasini et colleagues observed that CYP3A5 expressers required a higher TAC dose than nonexpressers, also showing a lower pre-dose concentration [32]. On the other hand, a lower dose in CYP3A*3/*3 carrier was reported by Thervet et al. [33] and by Yildirim and colleagues, who also found that dose-adjusted TAC concentration was statistically higher in the *3/*3 genotype 3 and 6 months post transplant (*p* < 0.05) [34]. Moreover, a Chinese study on a population of kidney transplant recipients found a much lower C_0_ in CYP3A5 expressers than in non-expressers (*p* < 0.01). However, no significant differences were found at 3 and 6 months post transplantation [35]. Furthermore, the target C_0_ (4–8 ng/mL) after initial dose was achieved less amongst expressers in comparison with non-expressers, and CYP3A5 non-expressers presented higher C_0_ (>8 ng/mL) 3 months post transplantation. A lower C0 in CYP3A5*1 carrier was also found in other studies carried out in different ethnic groups [36,37,38,39,40] (Table 1).

In addition, numerous papers evaluated the through concentration/dose ratio (C_0_/D), showing that it was lower in CYP3A5*1 carriers compared to wild-type [32,41,42,43,44,45,46] (Table 1). Moreover, Zhao and colleagues found that the weight-normalized oral clearance was lower in patients with CYP3A5*3/*3 genotype when compared to patients who were CYP3A5*1/*3 carriers [47]. On the other hand, Andrews et al., in a population of 337 kidney transplant recipients, observed that CYP3A5 expressers had a significantly higher TAC clearance, while CYP3A4*22 carriers had a significantly lower clearance [48], as confirmed by Zuo and colleagues [49] (Table 1).

Hannachi et al. showed a significant decrease of TAC C_0_/D in CYP3A4*1B carriers compared to wild-type, also demonstrating a higher C_0_/D ratio in patients with the CYP3A4*22 allele compared to non-carriers [50].

Furthermore, Lloberas et al. stratified 272 renal transplant recipients into poor metabolizers (PM, CYP3A4*22 + CYP3A5*3/*3), intermediate metabolizers (IM, CYP3A4*1/*1 + CYP3A5*3/*3 or CYP3A4*22 + CYP3A5*1), and extensive metabolizers (EM, CYP3A4*1/*1 + CYP3A5*1). Their results showed that dose-adjusted C_0_ was 88% lower in EM than IM and 26% higher in PM. Moreover, supra-therapeutic TAC exposure (C0 > 15 ng/mL) was significantly more frequent in PM than EM at 5–7 days after transplant (*p* = 0.01), and about 30% of EM had subtherapeutic exposure (C_0_ < 5 ng/mL) 5–7 days post transplant (*p* = 0.001) [51]. Min et al. reported that, 30 days after transplant, patients assigned to the genotype-guided arm reached therapeutic concentrations earlier than those in the standard-dosing arm, and they also had fewer out-of-range concentrations [52]. Similar results were obtained by Yanik et al.: in a population of 98 pediatric kidney transplant recipients observed, CYP3A5 expresser needed a significantly longer time to achieve a TAC-steady therapeutic concentration compared to non-expressers [53]. Furthermore, a study conducted in African American CYP3A5 expressers and non-expressers treated with once-daily formulation (LCPT-extended-released tacrolimus) or an immediate-release formulation (IR-Tac, immediate-release tacrolimus) showed that CYP3A5 expressers and nonexpressers did not have significant differences in AUC_0–24_ or C_min_ during administration of either IR-Tac or LCPT. They also found that tacrolimus C_max_ with IR-Tac or LCPT was 33% and 11% higher in CYP3A5 expressers than non-expressers [54]. Moreover, a retrospective study on 100 renal transplant recipients showed that CYP3A5 non-expressers had 63.3% of over-exposure (12 < C_0_ < 20 ng/mL) or 20.8% toxic concentrations (C_0_ ≥ 20 ng/mL). On the contrary, 25% of the heterozygote carriers showed overexposure contrary to none of the 1/*1 carriers. When new TAC starting doses of 0.10, 0.20, and 0.30 mg/kg/d were chosen for CYP3A5*3/*3, CYP3A5*1/*3, and CYP3A5*1/*1 genotypes, respectively, authors found that TAC overexposure was reduced in the CYP3A5*3/*3 group (*p* = 0.038), and none of the heterozygous patients presented toxic TAC C_0_ [55].

However, a few studies did not find any association between CYP3A polymorphisms and tacrolimus PK, as demonstrated by Shuker et al., who showed how adapting the tacrolimus starting dose to the different CYP3A5 genotypes does not increase the number of patients achieving therapeutic tacrolimus exposure early after transplantation and does not improve the clinical outcome in a population with low immunological risk [56]. Similar results were reported by Spierings et al. [57] and by Sienkiewicz et al. [58].

Nevertheless, inter- and intra-patient variability cannot be entirely explained with the presence of SNPs in genes encoding for cytochromes, and therefore, other genes must be involved, such as genes encoding for transporters. In this context, Ogasawara et al. investigated the influence of polymorphisms in ABCB1, ABCC2, and ABCG2 on dose-normalized (C_0_/D) TAC concentration. The authors observed a significant higher C_0_/D in carriers of the ABCC2 1249A allele and a lower C_0_/dose in patients with the ABCC2 3972T allele; on the other hand, they did not find any association between ABCB1 and ABCG2 genotypes and the C_0_/dose TAC concentration [59]. Another study on 91 kidney transplanted recipients demonstrated that patients with G2677T/A and C3435T SNPs in the MDR1 gene needed higher TAC doses than those required in the wild-type [20]. Accordingly, Provenzani et al. reported that kidney transplant recipient carriers of the 2677T/A allele needed a significantly higher daily tacrolimus dose compared with patients homozygous for the wild-type allele [60]. A lower TAC C0/D was also observed by Hu et al. in carriers of the ABCB1 61G allele [41] (see Table 1).

Moreover, a Chinese study investigated the associations between tacrolimus concentrations and SLCO1B1 polymorphisms in kidney transplant recipients, showing that TAC-dose-adjusted concentration was considerably higher in SLCO1B1 rs2306283 CC carriers compared with CT and TT carriers [61]. Furthermore, Boivin et al. investigated the association between T334G and G699A polymorphisms in the SLCO1B3 gene and TAC pharmacokinetics in renal transplant recipients. They found a 14.3-fold higher risk of over-exposure in carriers of the homozygous mutant haplotype (poor OATP1B3 transporters) compared to patients with heterozygotes or wild-type haplotype [62].

On the other hand, some studies found that polymorphisms in the ABCB1 gene did not have a significant influence on adjusted TAC trough concentrations or dose requirements [29,34,46] (Table 1).

Furthermore, a recent retrospective study assessed the variability in tacrolimus blood concentrations in 75 transplanted patients and investigated if tacrolimus blood levels were correlated with the compresence of several genetic polymorphisms: CYP3A5*1 (G6986A), CYP3A4*1B (A392G), CYP3A4*22, ABCB1 (C3435T; C1236T; G2677A/T), and SLCO1B1 (T521C). Based on the effect of their genotypes, patients were stratified into three groups: reduced tacrolimus metabolism (RM), increased metabolism (IM), and transporters polymorphisms (TM). Results showed that the percentage of patients with TAC levels out of therapeutic range was significantly higher in the IM group when compared with the WT or the TM group (*p* = 0.001 and *p* = 0.004). Moreover, an IM pattern resulted, as an independent predictor of number of tacrolimus blood levels out of therapeutic range (*p* = 0.015), while RM pattern was inversely related to the TAC administered dose (*p* = 0.006) [13].

In addition to evaluate the effects of SNPs on pharmacokinetics and pharmacodynamics parameters, we reported the results of several studies that also considered the clinical outcomes of the polymorphisms. In this context, Anutrakulchai et al. found that the genotype-guided group had more patients with tacrolimus concentrations in the therapeutic range and had also a lower proportion of over-therapeutic concentration. Surprisingly, they observed that delayed graft functions (DGFs) were more frequent in the genotype-guided group, whilst there were no significant differences of glomerular filtration rates and of graft or patient survivals during a median 37-month follow-up period [63]. Thervet et al. studied 280 kidney transplant recipients and found no differences in the incidence of delayed graft function (DGF) between genotype-guided group and control group [64]. Moreover, in a study on 136 de novo kidney transplant recipients, Hesselink et al. found that the frequency of BPAR (biopsy-proven acute rejection) between CYP3A5 expressers and nonexpressers was similar [36]. Furthermore, Roy et al. found there was no difference in the percentage of biopsy-confirmed acute rejection amongst the groups during the first 3 months post intervention in patients with less than three copies of MDR-1 (T-129C, C3435T, and G2677T) polymorphisms compared with patients having three or more copies of MDR-1 genetic variants [43]. Another study investigated the influence of CYP3A5 and ABCB1 SNPs on tacrolimus daily dose and transplantation outcomes on a population of on one hundred and thirty-six renal graft recipients and found that CYP3A5*1/*1 had an increased risk of acute rejection compared to CYP3A5*1/*3 and CYP3A5*3/*3 carriers. Instead, ABCB1 polymorphisms were not associated with transplantation outcomes [28]. A Japanese study on 41 renal allograft recipients reported that there was no difference in incidence of subclinical acute rejection between CYP3A5 expressers and wild-type. Moreover, the chronic allograft nephropathy (CAN) was more frequent in CYP3A5 expressers [39].

In summary, several studies including a total of 932 patients showed that higher tacrolimus doses are needed for patients carrying the CYP3A5*1 allele, and these results were confirmed by numerous other papers involving a total of 1302 patients that demonstrated a correlation between the presence of CYP3A5*1 allele and lower tacrolimus C_0_ levels and C_0_/D ratio; this suggests that a pharmacogenetic pre-evaluation could be useful for prescribing the most appropriate drug dose according to the patient’s genetic profile (Table 1).

### 3.2. Pharmacogenetics of Cyclosporine

Cyclosporine is one of the most used immunosuppressive agents after organ transplant, and several studies have explored the role of SNPs in cytochromes and transporters genes that might influence its pharmacokinetics. Cyclosporine PK is mainly affected by polymorphisms in CYP3A4, CYP3A5, and MDR1 genes. In fact, a study conducted by Żochowska and colleagues showed that patients with at least one functional CYP3A5*1 or CYP3A4*1B allele need significantly greater dosages of cyclosporine to reach target drug levels than patients with the CYP3A5*3 or CYP3A4*1 allele (*p* < 0.218) [65]. Meng et al. [66] and Qiu et al. [67] showed that CsA C_0_ and C_0_/D were significantly higher in patients carrying CYP3A5*3/*3 genotype than in patient carriers of A/A or G/A genotypes (Table 1). In another study, Li et al., in 83 renal-transplanted patients, found a statistical difference in the cyclosporine dose-adjusted 2 h post-dose concentrations (C_2_/D) between CYP3A4*1/*1 or CYP3A4*1/*18B carriers compared to the CYP3A4*18B/*18B group, while no difference was found in C_0_/D among the three genotypes [46]. Another study by Lunde et al. in 177 renal transplant patients during the early post-transplant period showed that CsA C/D was 53% higher among CYP3A4*22 carriers compared to WT (*p* = 0.03) [68]. Moreover, Kotowski et al., in a population of Polish kidney-transplanted patients, found that CYP3A4*1/*1 carrier received a lower mean dose of CsA and had a higher blood–drug concentration compared to CYP3A4*1/*1B. Regarding MDR1 3435C>T polymorphism, the authors observed that carriers of the C/C genotype received lower doses of CsA compared to patients with the C/T and T/T genotypes [69]. In another work on renal transplant recipients, Zhang et al. demonstrated that ABCB1 2677 T/T carriers had a significantly higher dose-adjusted trough concentration (C_0_) of CsA than G/G and G/T carriers (*p* = 0.001) in the early post-transplant period. Authors also found a significantly higher CsA C_0_ in ABCB1 3435 T/T carriers compared to C/C and C/T carriers (*p* = 0.002). In addition, significantly higher CsA C_0_/D was observed in patients with the ABCB1 1236TT-2677TT-3435TT haplotype compared to patients with other genotypes (*p* = 0.001) [70]. Moreover, Hu et al. demonstrated that the median cyclosporine dose-adjusted C_0_ in CYP3A5*1/*1 carriers was lower than CYP3A5*1/*3 and CYP3A5*3/*3 carriers during the early period post transplant. In addition, patients wild-type homozygotes for MDR1 C3435T had a slight but significantly lower dose-adjusted C0 than heterozygotes [71]. Yates et al. found that patients with at least one 3435T allele had a significantly higher CsA oral clearance than homozygous wild-type individuals [72]. On the other hand, Anglicheau et al. [73] and Sienkiewicz et al. [58] reported no correlation between the CYP3A5 genotype and CsA level. Another study found that dose-adjusted CsA C_0_ or C_2_ levels were not associated with CYP3A5, CYP3A4, and ABCB1 genotype. Moreover, the incidence of biopsy-proven acute rejection (BPAR) between the different ABCB1 genotype groups was comparable, and no significant difference in the incidence of BPAR was found between CYP3A5 expressers and nonexpressers or between CYP3A4*1 homozygote versus CYP3A4*1B carriers [74].

In summary, several papers involving a total of 410 patients demonstrated that the presence of the CYP3A5*1 and/or CYP3A4*1B allele is associated with higher cyclosporine dose and lower C_0_ and C_0_/D ratio levels. Instead, numerous other papers including a total of 384 patients showed that the presence of CYP3A4*22 allele, ABCB1 2677 T/T, and ABCB1 3435 T/T genotypes as well as the ABCB1 1236TT-2677TT-3435TT haplotype was associated with higher cyclosporine concentrations (C_0_/D and C_2_/D ratio), suggesting that a pharmacogenetic pre-evaluation could be useful for a targeted treatment of transplanted patients (Table 1).

### 3.3. Pharmacogenetics of Mycophenolic Acid

In solid organ transplant, mycophenolic acid (MPA) is used mainly as a CNI/sparing agent in order to diminish the dosage of these drugs and, by doing so, prevent their side effects. The most studied SNPs for the MPA are those involving ABCB2 and UGT family genes. In this regard, Fukuda et al., in 32 pediatric renal transplant recipients, observed that those heterozygous for MRP2-24T>C who also had UGT1A9-440C>T or UGT2B7-900A>G and MRP2-24T>C wild-type patients who carried both UGT1A9-440C>T and UGT2B7-900A>G presented a 2.2- and 1.7-times higher MPA dose compared to carriers of no UGT-SNPs (*p* < 0.001). In addition, a correlation between the presence of UGT1A9-440C>T allele and the inter-individual variability in peak concentrations was observed in the same population (*p* < 0.05) [75]. Moreover, a Chilean study showed that carriers of the UGT1A9-275A allele had lower AUC_0_–12h/MPA-D when compared with UGT1A9-275T carriers. Instead, MRP2 and UGT1A9 genotypes did not show significant differences in MPA C_0_, MPA-D, or MPA C_0_/D in 104 pediatric renal transplant recipients [76]. These results were confirmed by Mazidi et al. [77] (Table 1). Another study by Xie et al. showed that kidney-transplanted patient carriers of the UGT2B7 IVS1 + 985AG had a 48% higher dose-adjusted MPA AUC_0–12 h_ compared with IVS1 + 985AA carriers (*p* = 0.002). They also found a significantly higher dose-adjusted MPAG AUC_0–12 h_ in patents with the UGT1A9-1818CT rather than patients with UGT1A9-1818CC (*p* = 0.002). Moreover, UGT1A9-440C>T and -331T>C mutant carriers were correlated with an increase in MPAG AUC_0–12 h_ rather than wild-type (*p* = 0.028). In addition, carriers of UGT1A8*1/*1 had higher MPAG AUC_0–12 h_ compared to carriers of UGT1A8*1/*1 and UGT1A8*2/*2 (*p* = 0.004). Finally, patients with the UGT1A7 622TT genotype had lower MPAG AUC_0–12 h_ than UGT1A7 622CC (*p* = 0.012) [78]. Furthermore, Ciftci et al., in Turkish renal transplant recipients, showed that one month after transplant, patients carrying the UGT1A9 1399 T/T genotype had significantly higher MPA through blood concentrations and lower MPA doses compared to C/T and C/C carriers (*p* = 0.046 and *p* = 0.021, respectively) [79]. Furthermore, two papers by Kuypers et al. [80] and by Sánchez-Fructuoso et al. [81] found that patients with the T275A and C2152T SNPs of the UGT1A9 gene promoter had a significantly lower MPA exposure compared with patients who did not carry these mutations (Table 1).

On the other hand, only one study by Yang et al. that investigated several polymorphisms in different genes such as UGT1A8, UGT1A9, UGT2B7, ABCB1, ABCC2, ABCG2, SLCO1B1, and SLCO1B3 in 191 adult kidney-transplanted patients did not show an obvious impact of these genetic polymorphisms in metabolic enzymes and transporters on the PK of MPA [82].

In summary, some papers involving a total of 372 patients demonstrated that the presence of UGT1A9 T-275A polymorphism was associated with lower MPA concentrations, while a paper including 125 patients showed that the UGT1A9 1399 T/T genotype was related to higher MPA through blood concentrations and lower MPA doses; however, considering that few pharmacogenetic studies in transplanted patients are present in the literature, the role of SNPs affecting MPA pharmacokinetics should be further investigated. 

### 3.4. Pharmacogenetics of Everolimus and Sirolimus

Pharmacogenetics studies involving mTOR inhibitors have primarily focused their attention on the effects of SNPs in CYP3A4, CYP3A5, and ABCB1 genes [83]. A study conducted in 48 kidney transplant recipients treated with sirolimus (SIR) showed that in the early post-transplant period, carriers of CYP3A5*1/*3 presented lower SRL levels and level/dose ratio (LDR) than CYP3A5*3/*3 (*p* = 0.003 and *p* = 0.019, respectively). Regarding ABCB1 polymorphisms, sirolimus levels were higher in ABCB1 3435C>T with C/T genotypes than C/C and T/T in the late period (*p* = 0.038) [84]. Moreover, in a retrospective study, Lolita et al., in 69 renal-transplanted patients, found that the mean trough SRL concentration of patients with the CYP3A4 rs2242480 C/C genotype was significantly higher compared to the T/C and T/T group (*p* < 0.0001) [85]. Furthermore, Lee et al. observed lower SIR C_0_/D ratio in subject carriers of at least one CYP3A5*1 allele compared to patients with a homozygous CYP3A5*3 genotype (*p* < 0.05) in 85 Chinese renal-transplanted patients. On the other hand, no significant differences in SRL C_0_/D ratios were found between patient carriers of ABCB1 1236C>T, 2677G>T/A, and 3435C>T genotype compared to wild-type. In addition to this, haplotype analysis (which takes into consideration the combination of genetically associated SNPs) within the ABCB1 gene, including ABCB1 1236C>T, 2677G>T/A, and 3435C>T SNPs, showed that carriers of CGC/CGC diplotype had a mean SRL C_0_/D about 30% lower than carriers of CGC/TTT or TTT/TTT diplotype regardless of their CYP3A genotype (*p* < 0.05). These findings suggested that the haplotype of ABCB1 might be a better indicator for the prediction of SRL blood concentration than single SNP [86]. Furthermore, two studies found that carriers of CYP3A5*3/*3 had significantly higher SIR concentration/dose ratio (C/D), while polymorphisms in CYP3A4 and ABCB1 genes did not have a significant influence on adjusted SIR C/D or dose requirements [42,87] (Table 1). Tamashiro et al., in a study in 46 stable kidney transplant patients, found that the TAC C_0_/D was lower in CYP3A4 rs2242480, and both TAC and SRL C_0_/D were higher in CYP3A5 rs15524 TT carriers compared with CT and CC carriers. Moreover, patients with ABCB1 rs1045642 SNP and TT genotype had lower SRL C_0_/D at only 15 months after transplant [55]. Finally, only one study by Moes and colleagues investigated the prediction of EVE systemic exposure in renal transplant patients identifying the influence of a selection of SNPs in genes encoding for ABCB1, CYP3A5, and CYP2C8, but their results showed that all the selected polymorphisms had no clinically relevant effect on EVE pharmacokinetics [88].

In summary, some papers including a total of 272 patients demonstrated that the presence of CYP3A*1 allele was associated with lower sirolimus C_0_ and C_0_/D ratio levels, while the effects of SNPs in CYP3A4 and ABCB1 genes in transplanted patients under mTOR-inhibitor therapy should be further investigated (Table 1).

## 4. Discussion and Conclusions

Since the introduction of CsA in 1983 and Tac in 1989 for immunosuppressive therapy in transplanted patients, great progress in terms of graft survival has been made [89]. Further improvements have been achieved with the employment of ISDs not belonging to the CNI class, such as MPA, EVE, and SIR. Nonetheless, ISDs are aggravated by an important pharmacokinetic and pharmacodynamic inter- and intra-patient variability, and amongst the variables that influence ISDs’ PK and PD in transplanted patients, several studies have underlined the importance of the genetic characteristics of both the recipient and donor. In particular, the fundamental role of polymorphisms in genes encoding for enzymes and transporters responsible of the metabolism of the ISDs has been pointed out. Seeing that the reported survival rates of both allograft kidneys and transplanted patients increase when stable values in CNI trough level are achieved, it is important to take into consideration as many factors as possible when choosing the therapeutic regimen, including genetic factors that may influence the ISDs variability. For this reason, the association between SNPs and response to immunosuppressant therapy in transplants has been investigated by numerous studies, finding an association between some SNPs and the response to immunosuppressive therapy.

In this work, we reviewed the studies on the genetic polymorphisms associated with PK and PD variability in kidney-transplanted patients. Our results suggest that some SNPs are associated with the blood concentrations of immunosuppressant drugs in transplanted patients. Specifically, it has been observed that higher tacrolimus and cyclosporine doses are needed for patients carrying the CYP3A5*1 and/or CYP3A4*1B allele. Moreover, high cyclosporine blood levels were related to the presence of the CYP3A4*22 allele, ABCB1 2677 T/T and ABCB1 3435 T/T genotypes, as well as at ABCB1 1236TT-2677TT-3435TT haplotype (Table 2). In accordance with our findings, a recent review by Cheung and Tang highlighted that CYP3A5 expressers needed a higher tacrolimus dose, while the presence of CYP3A4*22 was associated with a lower tacrolimus dose requirement, and the combined CYP3A4 and CYP3A5 genotype can have a major influence on the tacrolimus dose required to reach the target exposure in kidney transplant recipients [90]. However, the authors did not assess the influence of several important SNPs on genes involved in immunosuppressant’s pharmacokinetics (ABCB1, UGT1A9, UGT2B7, MRP2). In regard to the correlation between tacrolimus levels and CYP3A5 SNPs, it is worthy of mention that in 2015, the Clinical Pharmacogenetics Implementation Consortium (CPIC) published guidelines for CYP3A5 genotypes and tacrolimus starting dose, where authors recommend that the poor-metabolizer phenotype should receive the standard dosing of ISD, while extensive and intermediate metabolizers should be administered with a 1.5–2-times higher starting dose [91].

Furthermore, our results showed that the presence of CYP3A*1 allele was associated with lower sirolimus C_0_ and C_0_/D ratio levels, while the effects of SNPs in CYP3A4 and ABCB1genes should be further investigated for these drugs.

Concerning the mycophenolic acid, it has been observed that the UGT1A9 T-275A polymorphism was associated with lower blood concentrations, while the UGT1A9 1399 T/T genotype was related to higher MPA through blood concentrations and lower MPA doses even if few pharmacogenetic studies were conducted in transplanted patients under MPA treatment, and therefore, the role of SNPs affecting MPA pharmacokinetics should be further investigated (Table 2). Despite these evidences, there is still not a definitive answer to the question of whether genotyping should be considered a standard practice in transplantation. This question is not easy to answer because of the multi-factorial approach adopted to assess a drug’s pharmacokinetic profile. In fact, genetic polymorphisms are only one of many factors that can influence ISDs’ PD and PK. Recipient age, race, body mass index, co-medication, but also donor age, graft functioning, and time since transplantation all play an important role in altering drug parameters. To further complicate the matter, studies in transplantation are often difficult to design because of the restricted patient population. Many studies are conducted on less than 100 patients, which may help elucidate some of the discordant results. Some of these studies also differ in patient characteristics, pharmacokinetic methods, times when blood drug concentrations are measured, and dosing strategies [92]. All the above-mentioned factors show that genotyping is a fascinating option when choosing the starting dose of ISDs. In addition, a patient’s genotype is stable and needs to be studied only once, unlike phenotypic characteristics that may change with environmental influences. Nevertheless, to definitively prove the helpfulness of genotyping, clinical studies must demonstrate that patients’ genotyping leads to a better use of a certain drug and to an improvement in that medication’s efficacy and safety. Additionally, given the elevated costs of genotypic tests and the well-known utility of therapeutic drug monitoring, not many researchers find genotyping transplant patients to be convenient. This may change in the immediate future, as more studies will show new data, and improvements in the genotyping methods will decrease the costs of these types of tests.

In conclusion, several studies showed that SNPs in enzymes and transporters influence ISDs’ pharmacokinetics; therefore, a genetic pre-evaluation of kidney-transplanted patients could be useful for prescribing a targeted treatment in order to improve the efficacy of the therapy and, at the same time, minimizing overexposure and toxicity. Nonetheless, further pharmacogenetics studies should be conducted to confirm the role of genotyping as a standard practice in transplantation.

## Figures and Tables

**Table 1:** Summary of studies on pharmacogenetics of immunosuppressants.

Study	Number of Patients	Drug	Gene	RefSNP	Clinical Effects
Cheung et al., 2019	86	Tacrolimus	CYP3A5	rs776746	Higher dose in CYP3A5 expressers
Allegri et al., 2019	20	Tacrolimus	CYP3A5	rs776746	Higher doses in CYP3A5*1/*1 and *1/*3 carriers
Mourad et al., 2005	85	Tacrolimus	CYP3A5	rs776746	Higher dose in CYP3A5*1 carrier
Quteineh et al., 2008	136	Tacrolimus	CYP3A5	rs776746	Higher dose in CYP3A5*1 carrier
Tada et al., 2005	28	Tacrolimus	CYP3A5	rs776746	Higher dose in CYP3A5*1 carrier
Tsuchiya et al., 2004	30	Tacrolimus	CYP3A5	rs776746	Higher dose in CYP3A5*1 carrier
Muller, 2020	43	Tacrolimus	CYP3A5	rs776746	Higher dose in CYP3A5*1/*1 and *1/*3 carriers
Gervasini et al., 2012	103	Tacrolimus	CYP3A5	rs776746	Higher dose in CYP3A5*1 carrier
Thervet et al., 2003	80	Tacrolimus	CYP3A5	rs776746	Lower dose in CYP3A5*3/*3 carrier
Yildrim et al., 2019	67	Tacrolimus	CYP3A5	rs776746	Lower dose in CYP3A5*3/*3 carrier
Chen et al., 2017	194	Tacrolimus	CYP3A5	rs776746	Lower C_0_ in CYP3A5 expressers
Hesselink et al., 2008	136	Tacrolimus	CYP3A5	rs776746	Lower C_0_ in carrier of at least one CYP3A5*1 allele
Zhang et al., 2005	118	Tacrolimus	CYP3A5	rs776746	Lower C_0_ in CYP3A5 expressers
Ferraresso et al., 2007	30	Tacrolimus	CYP3A5	rs776746	Lower C_0_ in CYP3A5 expressers
Satoh et al., 2009	41	Tacrolimus	CYP3A5	rs776746	Lower C_0_ in CYP3A5 expressers
Tirelli et al., 2008	26	Tacrolimus	CYP3A5	rs776746	Lower C_0_ in CYP3A5 expressers
Hu et al., 2018	165	Tacrolimus	CYP3A5	rs776746	Lower C_0_/D in CYP3A5 expressers
Li et al., 2015	112	Tacrolimus	CYP3A5	rs776746	Higher C_0_/D in CYP3A5*3/*3 carrier
Roy et al., 2006	44	Tacrolimus	CYP3A5	rs776746	Higher C_0_/D in CYP3A5*3/*3 carrier and lower C_0_/D in patients with less than three copies of MDR-1 polymorphisms.
			ABCB1	rs1045642	
			ABCB1	rs2032582	
			ABCB1	rs3213619	
Wang et al., 2020	406	Tacrolimus	CYP3A5	rs776746	Higher C_0_/D in CYP3A5*3/*3 carrier
Zhao et al., 2005	30	Tacrolimus	CYP3A5	rs776746	Higher C_0_/D in CYP3A5*3/*3 carrier
Li et al., 2013	83	Tacrolimus	CYP3A5	rs776746	Higher C_0_/D in carrier of haplotype GG
			CYP3A4	rs28371759	
Zhao et al., 2013	22	Tacrolimus	CYP3A5	rs776746	Lower clearance in CYP3A5*3/*3 carrier
Andrews et al., 2019	337	Tacrolimus	CYP3A5	rs776746	Higher clearance in CYP3A5 expressers and lower clearance in CYP3A4*22 carrier
			CYP3A4	rs35599367	
Zuo et al., 2013	161	Tacrolimus	CYP3A5	rs776746	Higher clearance in CYP3A5*1
Hannachi et al., 2021	80	Tacrolimus	CYP3A5	rs776746	Decreased C_0_/D in CYP3A4*1B and CYP3A5*1 carrier. Increased C_0_/D in CYP3A4*22 carrier
			CYP3A4	rs2740574	
			CYP3A4	rs35599367	
Yanik et al., 2019	98	Tacrolimus	CYP3A5	rs776746	Longer time to achieve a steady therapeutic concentration in CYP3A5*1 expresser. Higher incidence of early allograft rejection in CYP3A5*1 expressers
Spierings et al., 2013	118	Tacrolimus	CYP3A5	rs776746	Higher dose in CYP3A5 expressers
Ogasawara et al., 2013	102	Tacrolimus	ABCC2	rs3740066	Lower C_0_/D in ABCC2 3972T allele carrier
Kravljaca et al., 2016	91	Tacrolimus	ABCB1	rs1045642	Lower C_0_/D in CTT/TTT carrier
			ABCB1	rs1128503	
			ABCB1	rs2032582	
Provenzani et al., 2011	50	Tacrolimus	CYP3A5	rs776746	Lower C_0_/D in patients with one copy of the CYP3A5*1 allele
Liu et al., 2016	89	Tacrolimus	SLCO1B1	rs2306283	Higher C_0_ in CC carrier
Boivin et al., 2013	38	Tacrolimus	SLCO1B3	rs4149117	Higher risk of over-exposure in SLCO1B3 334G and 699A homozygous haplotype
			SLCO1B3	rs7311358	
Anutrakulcha et al., 2019	63	Tacrolimus	CYP3A5	rs776746	More patients with achieved therapeutic range and lower proportion of over-therapeutic concentration in the genotype-guided group
Thervet et al., 2010	280	Tacrolimus	CYP3A5	rs776746	C_0_ above the target range in CY3A5*3/*3 carriers and below the target in CYP3A5*1/*1 carrier
Quteineh et al., 2008	136	Tacrolimus	CYP3A5	rs776746	Higher dose in CYP3A5*1/*1 carrier. Increased risk of acute rejection in CYP3A5*1/*1
Żochowska et al., 2012	100	Cyclosporine	CYP3A5	rs776746	Higher dose in CYP3A5*1 or CYP3A4*1B carrier
			CYP3A4	rs2740574	
Meng et al., 2012	126	Cyclosporine	CYP3A5	rs776746	Higher C_0_ and C_0_/D in CYP3A5*3 G/G carrier
Lunde et al., 2014	177	Cyclosporine	CYP3A4	rs35599367	Higher C_2_/D in CYP3A4*22 carrier
Kotowski et al., 2019	184	Cyclosporine	CYP3A4	rs2740574	Lower dose in CYP3A4*1/*1
Zhang et al., 2013	101	Cyclosporine	ABCB1	rs1045642	Higher C_0_/D in ABCB1 2677 T/T carrier. Higher C_0_/D in ABCB1 3435 T/T carrier. Higher C_0_/D in ABCB1 1236TT-2677TT-3435TT haplotype compared to other genotypes
			ABCB1	rs1128503	
			ABCB1	rs2032582	
Hu et al., 2006	106	Cyclosporine	CYP3A5	rs776746	Lower C_0_/D in CYP3A5*1/*1 carrier. Lower C_0_/D in wild-type homozygotes for ABCB1 C3435T
			ABCB1	rs1045642	
Yates et al., 2003	19	Cyclosporine	ABCB1	rs1045642	Patients with at least one ABCB1 3435T allele had a significantly higher CsA clearance than homozygous wild-type
Fukuda et al., 2012	32	Mycophenolic acid	MRP2	rs717620	Higher dose in MRP2-24T>C heterozygous with also UGT1A9-440C>T or UGT2B7-900A>G and in MRP2-24T>C wild-type with both UGT1A9-440C>T and UGT2B7-900A>G
			UGT1A9	rs2741045	
			UGT2B7	rs7438135	
Krall et al., 2021	104	Mycophenolic acid	UGT1A9	rs6714486	Lower AUC_0–12_/D in UGT1A9-275A carrier
Mazidi et al., 2013	40	Mycophenolic acid	UGT1A9	rs6714486	Lower AUC_0–12_ and C_max_ in UGT1A9 275A carrier
Xie et al., 2015	127	Mycophenolic acid	UGT2B7	rs62298861	Higher AUC_0–12_ in UGT2B7 IVS1+985AG, UGT1A9-1818CT, UGT1A9-440C>T and -331T>C. UGT1A8*2 allele is related to lower AUC_0–12_ as well as the UGT1A7 622TT genotype
			UGT1A9	rs13418420	
			UGT1A9	rs2741045	
			UGT1A9	rs2741046	
			UGT1A8	rs1042597	
			UGT1A7	rs11692021	
Ciftci et al., 2018	125	Mycophenolic acid	UGT1A9	rs2741049	Higher C_0_ and lower doses in UGT1A9 1399 T/T carrier
Kuypers et al., 2005	95	Mycophenolic acid	UGT1A9	rs6714486	Lower exposure in T275A and C2152T carrier
			UGT1A9	rs17868320	
Sánchez-Fructuoso et al., 2009	133	Mycophenolic acid	UGT1A9	rs6714486	Lower exposure in UGT1A9 T-275A and C-2152T carrier
			UGT1A9	rs17868320	
Rodríguez-Jiménez et al., 2017	48	Sirolimus	CYP3A5	rs776746	Lower C_0_ in CYP3A5*1/*3 carrier. Higher C_0_ in ABCB1 3435 C/T carrier
			ABCB1	rs1045642	
Lolita et al., 2020	69	Sirolimus	CYP3A4	rs2242480	Higher C_0_ in C/C carrier
Lee et al., 2014	85	Sirolimus	CYP3A5	rs776746	Lower C_0_/D in CYP3A5*1 carrier
Li et al., 2015	43	Sirolimus	CYP3A5	rs776746	Higher C_0_/D in CYP3A5*3/*3. No correlation between SRL trough concentrations or dose requirements with CYP3A4 and ABCB1 SNPs
			ABCB1	rs1045642	
			ABCB1	rs1128503	
			ABCB1	rs2032582	
Miao et al., 2008	50	Sirolimus	CYP3A5	rs776746	Higher C_0_/D in CYP3A5*3/*3 carrier. No differences between C_0_/D and ABCB1 SNPs
Tamashiro et al., 2017	46	Sirolimus	CYP3A5	rs776746	Higher C_0_/D in CYP3A5 TT carrier.

**Table 2:** Summary of SNPs’ effects on immunosuppressants.

RefSNP	Drug	Clinical Effects
rs776746	Tacrolimus	Higher dose, lower C_0_, lower C_0_/D, higher clearance, higher risk of allograft rejection
rs776746	Cyclosporine	Higher dose, lower C_0_, lower C_0_/D
rs776746	Sirolimus	Lower C_0_, lower C_0_/D
rs2740574	Tacrolimus	Higher dose, lower C_0_/D
rs2740574	Cyclosporine	Higher dose
rs35599367	Tacrolimus	Higher C_0_/D, lower clearance
rs35599367	Cyclosporine	Higher C_2_/D
rs1045642	Cyclosporine	Higher C_0_/D
rs2032582	Cyclosporine	Higher C_0_/D
rs6714486	Mycophenolic acid	Lower exposure
rs17868320	Mycophenolic acid	Lower exposure

## Data Availability

Not applicable.
